# Performance Analysis of LSTM, GRU and Hybrid LSTM–GRU Model for Detecting GPS Spoofing Attacks

**DOI:** 10.3390/s26041111

**Published:** 2026-02-09

**Authors:** Umur Kuriş, Özgür Can Turna

**Affiliations:** 1 Cyber Security Analyst and Operator, Cyber Security Vocational School, Istanbul Technical University, 34485 Istanbul, Türkiye; umurkuris@itu.edu.tr; 2 Computer Engineering, Faculty of Engineering, Istanbul University-Cerrahpaşa, 34320 Istanbul, Türkiye

**Keywords:** GPS spoofing, unmanned aerial vehicles, LSTM, GRU, hybrid deep learning model

## Abstract

The exposure of Unmanned Aerial Vehicles (UAVs) to Global Positioning System (GPS) spoofing attacks constitutes a major cybersecurity challenge. In this work, we conduct a comparative performance analysis of LSTM, GRU, and sequential LSTM–GRU hybrid deep learning models for the detection of GPS spoofing attacks. The ‘UAV Attack’ dataset was preprocessed, and the 11 most significant features were selected using correlation and mutual information algorithms. The models were evaluated using a robust 5-fold cross-validation framework. A combination of 99.31% accuracy, 96.98% recall, and a 97.47% F1-score was achieved by the LSTM–GRU hybrid model, distinguishing it as the leading performer in the experimental study. The LSTM model achieved the highest precision, with a value of 98.49%. ROC curves and AUC values confirmed that the classification performance of all models was close to perfect for the simulated dataset. The findings indicate that deep-learning-based models incorporating the hybrid LSTM–GRU architectures provide an effective and reliable approach designed to identify GPS-spoofing threats affecting UAVs.

## 1. Introduction

Advances in autonomous systems enable many tasks to be performed either without human intervention or with minimal intervention. These systems, capable of processing sensory information and making decisions, are now being used in many applications that were previously considered challenging. UAVs represent a class of autonomous technologies and are equipped with a wide range of sensors including gyroscopes, accelerometers, LiDAR, thermal sensors, optical cameras, and GPS/GNSS receivers [1]. UAVs have been widely adopted in both civilian and military contexts, serving purposes including applications related to disaster response, airborne surveillance, target localization, aerial imaging, and search-and-rescue operations and agricultural applications.

The successful execution of UAV missions is largely dependent on reliable positioning and navigation information. At this point, GPS stands out as a critical technology that provides UAVs with high-precision position, speed, and time information [2]. With the aid of GPS, UAVs can follow predefined trajectories, maintain stable hovering positions even under challenging environmental conditions, and execute automated tasks with high accuracy. The integration of GPS technology into UAV systems has enhanced their operational capabilities while also improving efficiency, reliability, and overall effectiveness during mission execution [3]. Within DJI UAV systems, GPS constitutes a core component for navigation mode, maintaining position, enabling autonomous return, and enforcing airspace limitations [4]. However, the lack of encryption and authentication in civilian GPS signals renders the system vulnerable to a range of cyberattacks [2].

GPS receivers operate by processing weak satellite signals originating from orbital altitudes of roughly 20,000 km. As a result, they are highly vulnerable to signal spoofing. GPS spoofing refers to an attack in which attackers inject artificially generated GPS signals at power levels exceeding those of legitimate satellites, thereby manipulating the target receiver’s position solution [5]. This attack can lead to the takeover control of the UAV’s position–velocity–time (PVT) solution [6], thus causing the UAV to be misdirected, disrupting critical missions, leading to data loss, material damage, and even threatening human life [7,8]. In military contexts, inaccuracies in position estimation may result in the engagement of unintended targets [9]. Due to the lack of encryption, GPS signal integrity is susceptible to a range of malicious interventions, notably spoofing-based attacks, meaconing and jamming [10]. The use of low-cost and easily obtainable devices such as Software-Defined Radio (SDR) has made it considerably easier to carry out such attacks [11,12].

Various methods based on signal monitoring, mathematical modeling, control theory, cryptography, and, more recently, artificial intelligence have been proposed to enable the identification and mitigation of GPS-spoofing threats [13,14]. The limitations of conventional approaches have motivated researchers to explore machine learning and deep learning models for addressing this problem [15,16].

This work conducts an in-depth performance comparison focused on LSTM, GRU, and hybrid LSTM–GRU models in detecting GPS-spoofing attacks targeting UAVs and assesses the effectiveness of deep learning approaches in addressing this critical cybersecurity challenge. The study utilized the publicly available “UAV Attack” dataset. Following a comprehensive preprocessing and feature engineering process, the models were trained, and the results were analyzed in detail.

### 1.1. Related Work

The problem of GPS spoofing in UAVs has been approached through multiple detection and defense mechanisms reported across the literature. These approaches can be classified as signal processing, control theory, cryptography and more recently machine learning and deep-learning-based techniques. This section summarizes key studies that are closely related to the subject matter.

#### 1.1.1. Traditional Methods and Signal-Based Approaches

Early studies on GPS spoofing detection focused on signal processing and control theory-based methods. For example, Panice et al. [17] developed a detection solution based on the One-Class Support Vector Machine and emphasized the advantage of this lightweight, hardware-free solution being integrable into small UAVs. Similarly, Qiao et al. [4] proposed a vision-based method using the UAV’s monocular camera and Inertial Measurement Unit (IMU) sensor data and were able to detect attacks within an average of 5 s. Among traditional methods, techniques based on metrics such as signal-to-noise ratio, carrier phase, and pseudo-range are also widely used [18]. However, these methods can be inadequate against sophisticated spoofing attacks and may require additional hardware costs [13].

#### 1.1.2. Machine Learning-Based Approaches

In recent years, ML-based methods have emerged as an effective alternative for GPS spoofing detection. Manesh et al. [18] achieved high detection probability and low false alarm rate using an Artificial Neural Network (ANN)-based classifier that utilizes GPS signal characteristics such as false range and Doppler shift. Whelan et al. [19] reported F1 scores of up to 99% using a novelty-based approach with single-class classifiers. Feng et al. [20] processed GPS and IMU data using a Genetic Algorithm-XGBoost (GA-XGBoost) model and achieved 100% detection accuracy 1 s after the start of the attack. Traditional ML algorithms such as SVM have also been frequently applied in this field [16].

#### 1.1.3. Deep Learning and Hybrid Models

Deep learning models, particularly Recurrent Neural Network (RNN) architectures, have been widely adopted in this domain due to their effectiveness in handling time-series data. Wang et al. [21] rapidly and accurately detected UAV GPS spoofing attacks using an LSTM-based model. Xiao et al. [22] used RNNs to detect abnormal behaviors of UAVs and achieved high accuracy. Agyapong et al. [1] trained various deep learning models using a benchmark telemetry dataset and demonstrated that they could detect deviations caused by GPS spoofing with high accuracy.

Hybrid and ensemble models also offer promising results. Gasimova et al. [23] comparatively investigated three ensemble-based machine learning methods and reported that the stacking model outperformed the others across all evaluation metrics. Dang et al. [24] achieved over 97% accuracy under two base stations using a deep ensemble learning-based system. The hybrid use of LSTM and GRU models has demonstrated effectiveness across a broad range of application domains, including traffic prediction, integrated navigation, and stock price prediction, indicating its potential for UAV safety as well.

#### 1.1.4. Other Related Studies

Other important studies in literature include:

- Sedjelmaci et al. [25] introduced a hierarchical attack detection and response framework and reported a high detection rate accompanied by a low false positive rate.
- Gaspar et al. [26] developed a portable GPS spoofing system based on Software Defined Radio (SDR), demonstrating the vulnerability of commercial receivers.
- Arteaga et al. [27] demonstrated the exploitability of GPS vulnerability in a commercial UAV and highlighted the security advantages of military GPS.
- Nayfeh et al. [28] achieved a detection rate better than 92% with a detection time under a millisecond, demonstrating the real-time applicability of ML-based modeling.
- Eshmawi et al. [29] reported an accuracy of 99.74% by employing a stacking ensemble model that integrates support vector machines and convolutional neural networks.
- İşleyen and Bahtiyar [30] demonstrated that the XGBoost model is effective in accurately detecting fraud incidents.

#### 1.1.5. The Gap in the Literature and the Position of This Study

The existing literature demonstrates that machine and deep learning can be effectively utilized in GPS spoofing detection; however, a comprehensive comparison of different architectures (LSTM, GRU) and especially hybrid combinations of these architectures using the same dataset and features is limited. Furthermore, most studies focus on a specific model and do not provide performance comparisons across a wide range. This study aims to fill this gap in the literature by systematically evaluating the performance of LSTM, GRU, and LSTM–GRU hybrid models using the same dataset (UAV Attack) and a comprehensive preprocessing process. The use of a hybrid model has the potential to offer better prediction accuracy and lower error rates compared to LSTM or GRU models alone.

The primary objectives of this study are to (i) evaluate the effectiveness of deep learning architectures in detecting GPS spoofing, (ii) conduct a systematic comparative analysis of standalone LSTM and GRU models against a hybrid LSTM–GRU approach, (iii) identify the most critical sensory features for robust attack detection using information-theoretic selection methods, and (iv) ensure the generalizability and robustness of the findings through a 5-fold cross-validation framework.

## 2. Materials and Methods

### 2.1. Dataset and Preprocessing

In this study, the “UAV Attack”, a publicly available dataset created for the detection of cyber-attacks targeting UAVs, was utilized. The dataset consists of multidimensional time series data collected from both normal and attacked UAV flights. The dataset, which originally consists of 84 attributes (columns) and 3622 observations (rows), includes various system parameters such as the UAV’s attitude, global position, GPS position and local position.

A comprehensive preprocessing process was applied to the dataset to increase the efficiency and effectiveness of model training. In the first stage, correlation analysis was performed to understand the structure of the dataset and eliminate attributes containing unnecessary information. Attributes with high correlation (|r| > 0.84) with the target variable and attributes with low correlation (|r| ≈ 0) with a negligible relationship with the target variable were removed from the dataset. As a result of this process, the number of attributes was reduced to 35.

In the next step, mutual information feature selection algorithm was applied to further improve the model’s generalization performance and reduce computational complexity. This approach enables the modeling of non-linear dependencies between input features and the target outcome. As a result of the analysis, 11 features with the highest information gain for the target class were selected as the final dataset. To ensure unit consistency across all evaluated sensory features, the alt_y attribute was converted from millimeters (mm) into meters (m) by dividing its raw values by 1000. This standardization ensures that all altitude-related metrics are represented in meters (m), facilitating a more uniform input distribution for the deep learning models.

The definitions of these selected features are presented below (Table 1), based on an examination of the original source of the dataset and the relevant uORB (Micro Object Request Broker) message protocols:

These features focus on metrics critical for detecting GPS spoofing attacks, such as the UAV’s position, speed and the uncertainty or margin of error in these measurements. The final dataset obtained through preprocessing and feature selection was used in training and evaluating the deep learning models described in subsequent sections.

### 2.2. Method and Models Applied

In this study, three different deep learning models, whose effectiveness has been proven in the analysis of time series data to detect GPS spoofing on UAVs, were implemented: LSTM [31], GRU [32] and LSTM–GRU hybrid model, which is created by sequentially combining these two models.

Hybrid deep learning models aim to create a model that is more powerful and robust than what a single model could achieve by combining the strengths of different architectures. The proposed LSTM–GRU hybrid model in this study combines the superior ability of LSTM to model long-term dependencies with the computational efficiency of GRU.

The model is designed with a structure where the data flow sequentially passes through an LSTM layer first, followed by a GRU layer. The LSTM layer is responsible for capturing complex and long-term patterns in the input time series. The output of the LSTM layer is fed directly into the GRU layer. The GRU layer then processes the abstracted features from the LSTM layer to enable the model to make its final decision.

**Figure 1:**
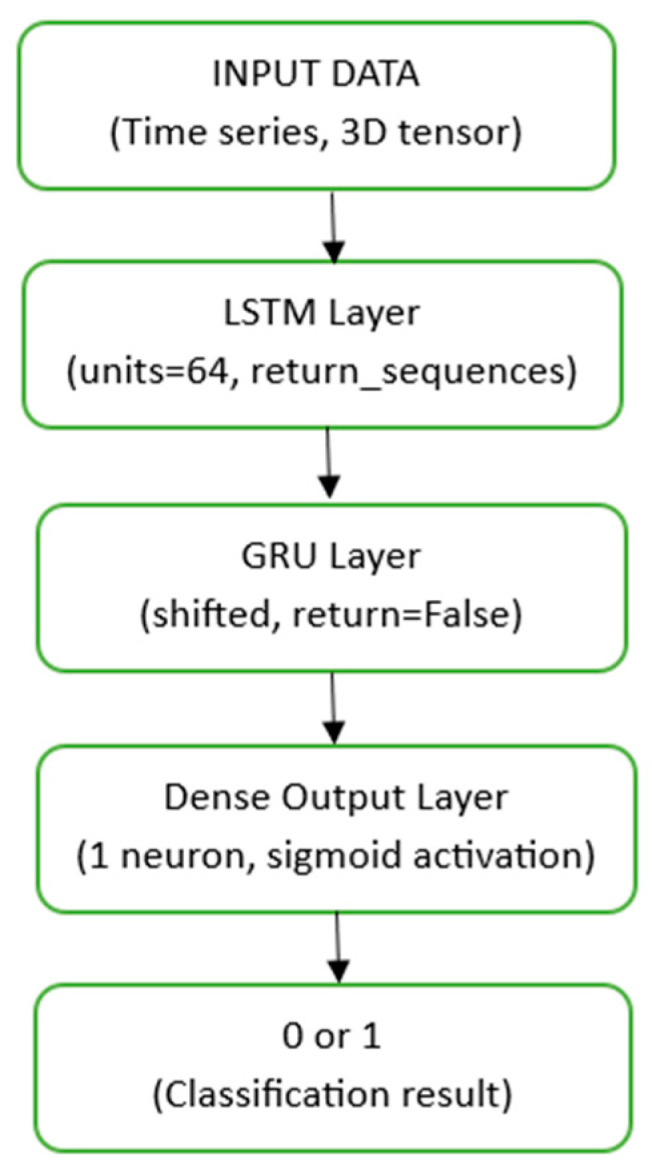
Schematic illustration of the LSTM–GRU hybrid architecture.

The theoretical basis of hybrid architecture is the synergistic effect produced by LSTM’s capacity to capture long-term dependencies and the GRU’s more compact structure and fast learning ability. These hybrid models have demonstrated successful application in several areas, including traffic flow prediction and financial time series analysis. Schematic illustration of the LSTM–GRU hybrid architecture applied in this study is given in Figure 1.

#### Model Training and Application Details

Python (version: 3.13), along with the TensorFlow/Keras frameworks, was employed to implement all proposed models. To ensure the statistical reliability and generalizability of the performance metrics, a 5-fold cross-validation framework was employed instead of a single static split. This approach ensures that the reported results represent the mean performance across the entire dataset, mitigating potential biases related to data partitioning. The models were configured to address a binary classification problem (attack versus normal). The output layer utilized a sigmoid activation function, while binary cross-entropy was selected as the loss function for model optimization. The Adam algorithm was used for optimization. Early stopping and regularization techniques were applied to prevent overfitting of the models. Early stopping was applied to all models, terminating training when no improvement in validation loss was observed over a specified number of epochs. The optimal number of layers, number of cells and learning rate values were determined for models.

To ensure the reproducibility of the experimental results, the specific hyperparameters and training configurations used for all evaluated models are summarized in Table 2. These settings were kept consistent across standalone and hybrid architectures to maintain a fair performance comparison.

As detailed in Table 2, the input sequence length was set to 1. This choice was primarily driven by the need for low-latency detection in UAVs, where identifying an attack in its earliest stages is critical for flight stability and safety. By processing data at each time step independently, the models can provide near-instantaneous classification outcomes without the computational delay associated with larger temporal windows. Furthermore, the high discriminative power of the selected 11 sensory features allows for high-accuracy detection even at this minimal sequence length.

## 3. Results

Among the evaluated models, hybrid LSTM–GRU model demonstrated the most robust performance across the 5-fold cross-validation, achieving a mean accuracy of 99.31%, a superior recall of 96.98% and F1 score 97.47%. The highest precision was observed in the LSTM model (98.49%). Table 3 reports accuracy, precision, recall, and F1-score results obtained from models evaluated using 11 features.

The number of trainable parameters for each architecture was calculated to assess the trade-off between performance and computational complexity. The standalone LSTM and GRU models consist of approximately 19,700 and 14,800 parameters, respectively. The hybrid LSTM–GRU model, which demonstrated the highest performance, possesses approximately 44,500 parameters. This increased capacity justifies its enhanced ability to model complex temporal dependencies and achieve a superior recall of 96.98%.

**Figure 2:**
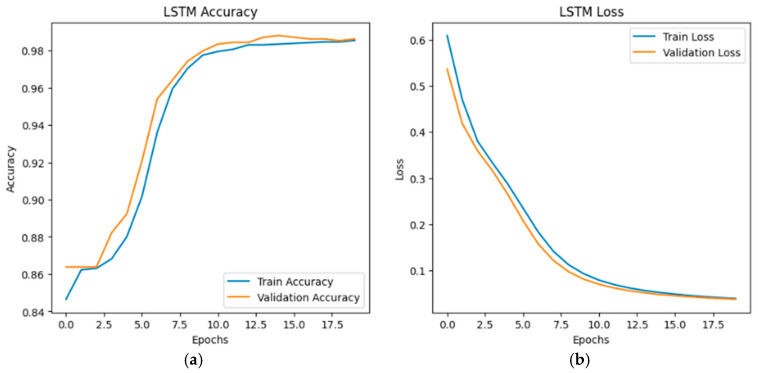
LSTM accuracy-loss graphs: (**a**) LSTM accuracy graph; (**b**) LSTM loss graph.

**Figure 3:**
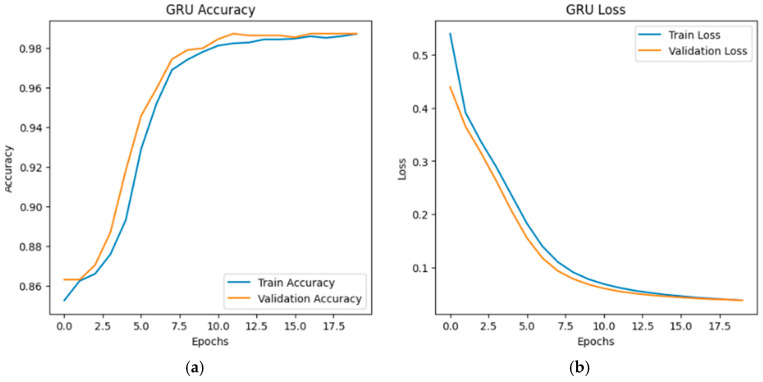
GRU accuracy-loss graphs: (**a**) GRU accuracy graph; (**b**) GRU loss graph.

**Figure 4:**
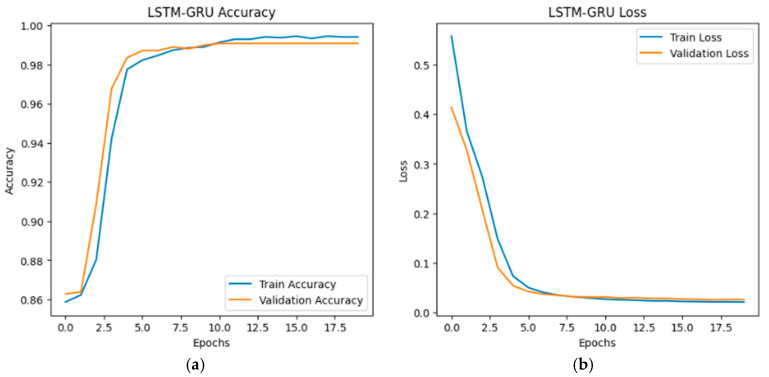
LSTM–GRU accuracy-loss graphs: (**a**) LSTM–GRU accuracy graph; (**b**) LSTM–GRU loss graph.

The training and validation accuracy and loss curves for the LSTM, GRU, and hybrid LSTM-GRU models are illustrated in Figure 2, Figure 3 and Figure 4, respectively. According to the graphs, all three models show similar learning curves; accuracy increased and loss decreased as epochs progressed. The LSTM–GRU hybrid model converged faster, reaching higher accuracy levels around the 5th epoch (see Figure 4a). All models stabilized at an accuracy level of 98–99% around the 15th epoch. Test accuracy (orange line) occasionally exceeded training accuracy (blue line), indicating good generalization ability.

**Figure 5:**
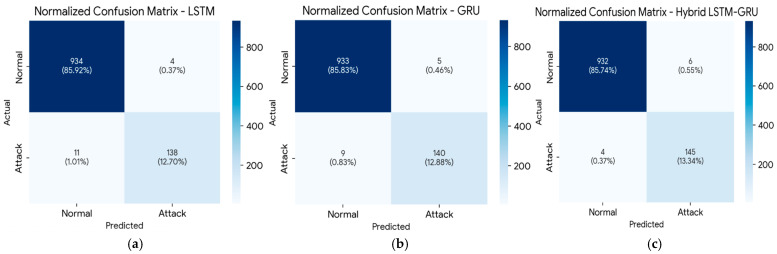
Normalized confusion matrices. Results represent a representative fold of the 5-fold cross-validation process, aligning with the reported mean performance metrics: (**a**) LSTM confusion matrix; (**b**) GRU confusion matrix; (**c**) LSTM-GRU confusion matrix.

**Figure 6:**
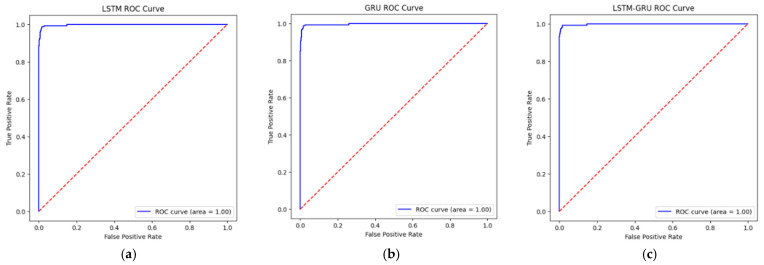
ROC curves: (**a**) LSTM ROC curve; (**b**) GRU ROC curve; (**c**) LSTM–GRU ROC curve.

Confusion matrices are used to depict the distributions of true and false classification outcomes for each model. The LSTM–GRU hybrid model showed the lowest false negative rate, but it has a slightly higher false positive rate (see Figure 5c). The classification performance and the distribution of true versus false outcomes for each model are depicted in the confusion matrices presented in Figure 5:

The three ROC curves in Figure 6 show the classification performance of the compared LSTM, GRU and LSTM–GRU hybrid models.

ROC curves validate our previous analyses and show that all models perform very well but the LSTM–GRU hybrid model has a slight advantage. The fact that the models achieve an AUC value of 1.00 indicates excellent classification performance on the utilized simulated dataset. This near-perfect separability is attributed to the distinct patterns of GPS spoofing attacks within the dataset generated via PX4/Gazebo simulations, which facilitate almost flawless classification under controlled conditions.

The results obtained indicate that the hybrid LSTM–GRU model is preferable, especially in applications requiring high accuracy.

## 4. Discussion

Our findings are consistent with the benchmark study by Whelan et al. [19], who introduced the ‘UAV Attack’ dataset. While Whelan et al. utilized one-class classifiers like Autoencoders to achieve high F1-scores, our research demonstrates that a supervised hybrid LSTM–GRU approach also yields robust results (97.47% F1-score) by effectively leveraging temporal dependencies. Unlike the PCA-based dimensionality reduction used in previous work, our study emphasizes the physical interpretability of individual sensory features. Additionally, the near-perfect AUC values observed in our tests align with the high separability of spoofing signals in simulated PX4/Gazebo environments, as also noted in [19].

Drawing on the outcomes of this study, the following recommendations are provided to guide future research efforts:

- Examination of different feature selection methods: In this study, 11 features were selected using the mutual information method. The effect of different feature selection or dimension reduction techniques (e.g., PCA, LDA, model-based importance scores) on performance can be investigated.
- Deepening hyperparameter optimization: To further improve model performance, more comprehensive hyperparameter optimization (e.g., Bayesian optimization) can be performed and parameters (e.g., optimal configuration of layers, cell counts and learning rate) can be investigated in greater detail.
- Validation with larger and more diverse datasets: To test the model’s generalization ability more robustly, work can be done on training and evaluating it on larger and more diverse datasets from different UAV platforms and containing different attack scenarios.
- Validation Strategy Limitations: It is important to acknowledge that the 5-fold cross-validation employed in this study was performed at the sample level rather than using a time-aware or flight-wise split. While sample-level splitting provides statistical robustness regarding feature space distribution, it may result in high performance due to the temporal correlation between adjacent samples in time-series data. In real-world operational scenarios, detecting attacks on completely unseen flight trajectories (flight-wise validation) represents a stricter generalization challenge. Future studies should prioritize flight-wise splitting strategies to rigorously evaluate the model’s performance on unseen trajectories and minimize potential data leakage arising from temporal proximity.
- Real-time application and deployment: Work can be done on optimizing the developed model to run in real time and integrating it as an embedded system on a UAV flight controller or ground station. In this context, model pruning, quantization, and latency analysis are critical.
- Research on different deep learning architectures: In future work, the performance of other advanced architectures, such as Transformer-based models or Convolutional Neural Networks for Long-Term Patterns (CNN-LSTM), can be investigated.
- Extension to other attack types: In addition to the GPS spoofing attack focused on this study, the development of similar deep-learning-based methods for detecting other cyber-attacks, such as communication channel eavesdropping, DoS and command injection, could be investigated.

In conclusion, this study has demonstrated that deep learning models, and hybrid approaches in particular, offer an effective solution for ensuring the cybersecurity of UAVs. Future work in the suggested directions will significantly contribute to the maturation of these technologies and their integration into real-world applications.

## 5. Conclusions

The performance of LSTM, GRU and hybrid LSTM–GRU models in detecting GPS-spoofing attacks targeting UAVs is examined in this study. Experimental studies conducted on the “UAV Attack” dataset yielded the following results:

- Hybrid model demonstrated superior performance: Our findings confirm that the hybrid architecture provides an optimal balance for UAV security, with an F1-score of 97.47%. Specifically, it minimizes missed detections (False Negatives) by reaching a recall of 96.98%, a significant improvement over single-layer recurrent models.
- LSTM provided high precision: The LSTM model demonstrated the highest positive predictive consistency, achieving a precision value of 98.49%. This indicates that the LSTM model exhibits superior reliability in its positive class predictions.
- All models achieved high success: ROC curves and 1.00 AUC values confirmed that all three models performed near-perfectly in distinguishing GPS spoofing attacks from normal conditions. This result proves that deep learning models are effective tools in the field of UAV cybersecurity.
- The hybrid model converged faster: During the training process, the hybrid model was observed to reach high accuracy values at earlier epochs (Figure 4). This indicates that the hybrid model also offers advantages in terms of learning efficiency.

These results demonstrate that the hybrid use of LSTM and GRU architectures is an extremely promising approach, particularly in critical applications requiring high accuracy and reliability, such as the detection of GPS spoofing attacks.

## Figures and Tables

**Table 1:** Descriptions of attributes used in the final data set.

Feature Name	Description	Unit	MI Score
*alt_ellipsoid_x*	Fused altitude relative to the ellipsoidal reference surface	meter (m)	0.315998
*epv_x*	Primary standard deviation of estimated vertical position error	meter (m)	0.274125
*alt_y*	Raw GPS altitude above Mean Sea Level (MSL)	meter (m)	0.284894
*alt_ellipsoid_y*	Raw GPS altitude relative to the ellipsoidal reference surface	meter (m)	0.284961
*s_variance_m_s*	GPS speed accuracy estimate (speed variance)	meter/second (m/s)	0.349014
*epv_y*	Vertical Dilution of Precision (VDOP) representing geometric quality	meter (m)	0.293474
*vdop*	Vertical Dilution of Precision (unitless geometric multiplier)	-	0.269708
*vel_m_s*	GPS ground speed	meter/second (m/s)	0.284417
*vel_d_m_s*	GPS downward velocity component (vertical speed)	meter/second (m/s)	0.264995
*cog_rad*	Course Over Ground	radian (rad)	0.281952
*epv*	System-wide vertical position error estimate (redundant check)	meter (m)	0.256721

**Table 2:** Detailed Training Hyperparameters and Configuration.

Parameter	Value/Detail
Input Sequence Length	1
Batch Size	64
Optimizer	Adam
Initial Learning Rate	0.001
Loss Function	Binary Cross-Entropy
Callbacks	Early Stopping (patience 3), ReduceLROnPlateau
Maximum Epochs	20

**Table 3:** Models’ Performance.

	Accuracy	Precision	Recall	F1
LSTM	0.9867	0.9849	0.9176	0.9501
GRU	0.9870	0.9829	0.9217	0.9513
LSTM-GRU	0.9931	0.9798	0.9698	0.9747

## Data Availability

Publicly available datasets were analyzed in this study. This data can be found here: https://doi.org/10.21227/00dg-0d12.

## Abbreviations

The following abbreviations are used in this manuscript:

UAV	Unmanned Aerial Vehicle
GPS	Global Positioning System
GNSS	Global Navigation Satellite System
IMU	Inertial Measurement Unit
uORB	Micro Object Request Broker
SDR	Software Defined Radio
ANN	Artificial Neural Network
RNN	Recurrent Neural Network
LSTM	Long Short-Term Memory
GRU	Gated Recurrent Unit
ROC	Receiver Operating Characteristic
AUC	Area Under the Curve
PVT	Position–Velocity–Time
